# Genetic and Functional Role of TNF-alpha in the Development *Trypanosoma cruzi* Infection

**DOI:** 10.1371/journal.pntd.0000976

**Published:** 2011-03-08

**Authors:** Cristina Wide Pissetti, Dalmo Correia, Rafael Faria de Oliveira, Maurício Manoel Llaguno, Marly Aparecida Spadotto Balarin, Roseane Lopes Silva-Grecco, Virmondes Rodrigues

**Affiliations:** 1 Laboratory of Immunology, Universidade Federal do Triângulo Mineiro, Uberaba, Minas Gerais, Brazil; 2 Department of Internal Medicine, Universidade Federal do Triângulo Mineiro, Uberaba, Minas Gerais, Brazil; 3 Laboratory of Genetics, Universidade Federal do Triângulo Mineiro, Uberaba, Minas Gerais, Brazil; University of Massachusetts Medical School, United States of America

## Abstract

TNF-alpha plays an important role in trypanocidal mechanisms and is related to tissue injury. This cytokine has been detected in the heart of human chagasic patients where it is associated with tissue damage. This study investigated whether TNF-alpha levels and the presence of genetic polymorphisms are associated with the presence of *T. cruzi* infection and/or with the development of the cardiac form in chronic chagasic patients. Genomic DNA of 300 subjects from an endemic area was extracted and analyzed by PCR using specific primers. TNF-alpha was assayed in culture supernatants by ELISA. An association was observed between the absence of the TNF-238A allele and negative serology. Furthermore, seropositive individuals carrying the TNF-238A allele produced significantly higher TNF-alpha levels without stimulation (p = 0.04) and after stimulation with LPS (p = 0.007) and *T. cruzi* antigens (p = 0.004). The present results suggest that the polymorphism at position -238 influences susceptibility to infection and that this allele is associated with higher TNF-alpha production in seropositive individuals.

## Author Summary

Chagas disease is an important parasitic disease that has no cure. The pathogenesis of the disease is still not completely understood. Studies using candidate genes are important to better understand the differences between individuals that lead to such heterogenous disease. TNF-alpha is a cytokine involved in the control of parasitemia during the acute phase and in cardiac injury during the chronic phase. The TNF-alpha gene is located in an important region of the MHC and its polymorphisms are associated with many parasitic and infectious diseases such as cerebral malaria and leishmaniasis. These studies are important since they were conducted in the same regions and involved populations leaving in the same conditions. The present study shows that patients with the indeterminate form produce higher levels of TNF-alpha than cardiac patients. The data suggest a control mechanism between inflammatory and regulatory cytokines. In addition, the presence of the TNF-238G allele contributes to the development of negative serology. We show that CD8^+^ T lymphocytes and macrophages are the main cells producing TNF-alpha. This study is an important contribution to explain the pathogenesis of Chagas disease.

## Introduction

Chagas disease is an important chronic infection caused by the protozoan *Trypanosoma cruzi*. The disease continues to be a major public health problem in most Latin America countries, affecting around 9 million people [1]. In addition, Chagas disease is an emerging health problem in non-endemic areas because of the increasing migration of individuals [2]. Chagas disease has two successive phases. The acute phase is usually asymptomatic or characterized by the presence of fever, discomfort, tachycardia and high parasitemia [3], [4]. Manifestations of the acute disease resolve spontaneously in about 90% of infected individuals even if the infection is not treated with trypanocidal drugs. About 60–70% of these patients will never develop clinically apparent disease. These patients have the indeterminate form of chronic Chagas disease, which is characterized by positivity for antibodies against *T. cruzi* in serum, a normal 12-lead electrocardiogram (ECG), and normal chest, esophagus and colon exams. The remaining 30–40% of patients will subsequently develop a determinate form of chronic disease: cardiac, digestive (megaesophagus and megacolon), or cardiodigestive [2].

Geographic variations exist in the severity and prevalence of the clinical forms of Chagas disease, but the reasons for this clinical and epidemiological heterogeneity are unknown [5]. Possible causes include variations in the genetic constitution of the host, especially genes related to the immune system since the latter is involved in the control of parasitism and in tissue injury. In Chagas disease, like in other parasitic diseases, the causal factor (i.e., the infectious agent) is necessary but often insufficient for clinical manifestation of the disease [6]. The complexity of the host-parasite relationship in Chagas disease suggests the involvement of different components of the immune system.

The genes for tumor necrosis factor-alpha (TNF-alpha) and lymphotoxin-alpha, which are located in the MHC III region on chromosome 6, are closely linked to the HLA class I and class II genes [7]. TNF-alpha, which is mainly produced by monocytes and activated T cells, plays an important immunoregulatory role [8]. This cytokine contributes to the pathogenesis of Chagas disease due to its role in both trypanocidal mechanisms and tissue injury. Production of this cytokine at high levels has been demonstrated in experimental models during the acute phase [9] and its function as an inducer of iNOS is important for the control of parasite growth [10], [11]. TNF-alpha has been detected in the heart tissue of experimentally infected animals [12], as well as in inflammatory exudate cells of human chagasic myocarditis [13]. These findings suggest that individual differences in TNF-alpha production may be responsible for the variation among individuals. These differences are the result of polymorphisms present in the general population. Polymorphisms in the promoter region of the TNF-alpha gene have been known for a long time and might be involved in the control of the expression of this gene [14], [15]. The presence of these polymorphisms has been associated with susceptibility to certain inflammatory and infectious diseases [16]–[18].

Recent studies have investigated the role of TNF-alpha gene polymorphisms in Chagas disease [19]–[22], but none of those studies evaluated a population from an endemic area or used the clinical assessment criteria established here. The present study investigated TNF-alpha production by peripheral blood cells and whether the presence of G substitutions at positions -238 and -308 are associated with the presence of *T. cruzi* infection and/or the development of the cardiac clinical form in chronic chagasic patients.

## Materials and Methods

### Study area

The study was performed in the municipality of Água Comprida (20°3′23″S, 48°6′31″W at 540 m above sea level), situated in the Vale do Rio Grande, southern region of Triângulo Mineiro, Minas Gerais State, Brazil. The town was endemic for Chagas disease and was included in the first National Campaign against *T. cruzi* that started in 1975. Epidemiological and entomological data demonstrated the interruption of vector transmission of the parasite to humans in 1999, and an international commission certified Brazil to be free of transmission in 2005 [23], [24].

### Study population

A total of 300 unrelated individuals agreed to participate in the study. Only subjects who were 25 years or older were included (mean age: 51.2±14.4; range: 25–91 years), since they corresponded to the youngest seropositive individuals of the sample studied. Serological screening for anti-*T. cruzi* antibodies showed that 25.6% of these subjects were positive and 74.5% were negative [25]. All HIV-seropositive individuals were excluded from the study. The treatment criteria did not exclude any patient.

### Ethics statement

The study was approved by the Research Ethics Committee of Universidade Federal do Triângulo Mineiro, Brazil (protocol 343). All individuals provided written informed consent.

### Serological test

The presence or absence of *T. cruzi* infection was evaluated by passive hemagglutination (Salck Laboratory, São Paulo, Brazil), enzyme-linked immunosorbent assay (Abbott, Brazil) and indirect immunofluorescence using fluorescein isothiocyanate (FITC)-conjugated to rabbit anti-human IgG (Sigma). The assays were performed according to manufacturer instructions and the results are expressed quantitatively. A subject who presented at least two positive tests was defined as positive [25].

### Characterization of clinical forms

Patients infected with *T. cruzi* were submitted to clinical examination, electrocardiography and chest, esophagus and colon contrast X-ray exams for classification into the cardiac, digestive, mixed, or indeterminate form [26], [27]. Patients with the cardiac form were classified according to the Criteria Committee of the New York Heart Association [28]. Appropriate statistical analysis was not possible because of the small sample size.

### Blood collection and cell culture

Peripheral blood samples (20 mL) were collected with a vacuum system using heparin as anticoagulant. Peripheral blood mononuclear cells (PBMC) were separated by density centrifugation on a Ficoll-Hypaque gradient (Pharmacia) according to manufacturer recommendations. After separation, the cells were washed by centrifugation in RPMI medium (Gibco) and resuspended in RPMI medium supplemented with 5% fetal bovine serum (Gibco), 2 mM L-glutamine (Gibco), 50 mM 2-mercaptoethanol (Merck), and 40 µg/mL gentamicin. PBMC (2×10^6^ cells/well) were cultured in the presence of 5 µg/mL *T. cruzi* (strain Y) antigens, 2 µg/mL *Salmonella typhimurium* lipopolysaccharide (LPS) (Sigma), and 5 µg/mL phytohemagglutinin (PHA) (Sigma). The plates were incubated at 37°C in a 5% CO_2_ atmosphere for 48 h. The culture supernatants were collected and stored at −70°C.

### Quantification of TNF-alpha by ELISA

For TNF-alpha titration, microplates (Nunc) were sensitized overnight with anti-TNF-alpha mAb (Pharmingen). Nonspecific binding was prevented by incubating the plates with 2% BSA (Sigma) in PBS. The plates were incubated overnight with 100 µL of the culture supernatants in PBS diluted 1∶2, 2% BSA, and recombinant human TNF-alpha (Pharmingen). The plates were then washed four times with PBS and 0.05% Tween 20 and incubated with biotinylated anti-TNF-alpha mAb (Pharmingen) for 2 h, followed by washing and incubation for 2 h with streptavidin-conjugated alkaline phosphatase. Finally, the plates were washed four times and enzymatic activity was developed by incubating the plates with *p*-nitrophenyl phosphate (Sigma). Absorbance was read at 405 nm in a microplate reader (BioRad). The sensitivity limit of the test was 10 pg/mL.

### Genomic DNA purification

The mass of red and white blood cells was submitted to osmotic lysis using Tris-EDTA lysis buffer (20∶5) consisting of 1 M Tris-HCl and 0.5 M EDTA, pH 8. The samples were centrifuged at least three times at 1334×*g* for 15 min at controlled room temperature (approximately 27°C).

For DNA extraction, the samples were treated by two different techniques depending on the amount of red blood cells remaining in the leukocyte pellet to guarantee the best quality DNA. A commercially available kit (DNAzol, Gibco) was used for samples containing few red blood cells and phenol-chloroform extraction was used for samples containing higher amounts of red blood cells. The samples were resuspended in water and DNA was analyzed by 1% agarose gel electrophoresis.

### PCR assays

Polymorphisms in the promoter region of the TNF-alpha gene were determined by PCR amplification, followed by digestion with appropriate restriction enzymes. For TNF-238G/A [15], PCR was carried out in a volume of 30 µL containing 11.70 µL sterile and filtered Milli-Q water, 3.0 µL 10× buffer without MgCl_2_ (Gibco), 1.0 µL 50 mM MgCl_2_ (Gibco), 3.0 µL 2 mM dNTPs (Invitrogen), 3.0 µL of each primer (40 nmol 238F: 5′ GGT CCT ACA CAC AAA TCA GT 3′, and 43.2 nmol 238R: 5′ CAC TCC CCA TCC TCC TCC CTG GTC 3′) (Gibco), 0.30 µL (500 units, 5 U/µL) Taq DNA polymerase (Gibco), and 5.0 µL genomic DNA at a concentration of 20 µg/mL. The following PCR conditions were used: 5 min at 95°C for initial denaturation and 35 cycles at 95°C for 1 min (denaturation), 55°C for 45 s (primer annealing), and 72°C for 45 s (extension), followed by a final extension step at 72°C for 3 min. Next, RFLP was performed using 0.1 µL *Ava*II (10,000 U/mL) (New England Biolabs), 1.0 µL NE 4 buffer provided with the restriction enzyme, 0.4 µL sterile and filtered Milli-Q water, and 8.5 µL of the PCR product in a final volume of 10 µL. The samples were incubated for approximately 18 h at controlled room temperature (approximately 27°C). The digestion products were analyzed on 12% polyacrylamide gel (37∶5∶1) stained with 1% silver nitrate. Digestion of the PCR products from patients homozygous for the TNF-238A allele (-238A/A) generated only one 71-base pairs (bp) fragment, whereas those from patients homozygous for the TNF-238G allele (-238G/G) were completely digested (51 and 20 bp). All three fragments (71, 51 and 20 bp) were present in heterozygous patients.

For TNF-308G/A [14], the PCR mixture contained 11.70 µL sterile and filtered Milli-Q water, 3.0 µL 10× buffer without MgCl_2_ (Gibco), 1.0 µL 50 mM MgCl_2_ (Gibco), 3.0 µL 2 mM dNTPs (Invitrogen), and 3.0 µL of each primer (38 nmol -308F: 5′ AGG CAA TAG GTT TTG AGG GCC AT 3′, and 45 nmol -308R: TCC TCC CTG CTC CGA TTC CG 3′) (Gibco), 0.30 µL (500 units, 5 U/µL) Taq DNA polymerase (Gibco), and 5.0 µL genomic DNA at a concentration of 20 µg/mL in a final volume of 30 µL. The following PCR conditions were used: 5 min at 95°C for initial denaturation, followed by 35 cycles at 95°C for 1 min (denaturation), 52°C for 45 s (primer annealing), and 72°C for 45 s (extension), followed by a final extension step at 72°C for 3 min. For RFLP, the mixture contained 0.1 µL *Nco*I (10 U/µL) (Gibco), 1.5 µL REACT 3 buffer provided with the restriction enzyme, 10.5 µL sterile and filtered Milli-Q water, and 3.0 µL of the PCR product in a final volume of 12.1 µL. The samples were incubated for approximately 18 h at 37°C. The digestion products were analyzed on 10% polyacrylamide gel (37∶5∶1) stained with 1% silver nitrate. Digestion of the PCR products from patients homozygous for the TNF-308A allele generated only one 107-bp fragment, whereas those from patients homozygous for the TNF-308G allele were completely digested (87 and 20 bp). All three fragments (107, 87 and 20 bp) were present in heterozygous patients.

### Flow cytometry immunostaining and data acquisition

The following antibodies purchased from BD Pharmingen were used: FITC anti-CD8, FITC anti-CD14, and PE-Cy5 anti-CD4. PE anti-TNF-alpha and appropriate isotype controls were used. All of these antibodies were used according to manufacturer instructions. PBMC were recovered from 48 h cell cultures in the presence of *T. cruzi* antigens or medium alone. The cells were transferred (2×10^5^ cells/tube) to 5 mL polystyrene tubes (Falcon®) and washed once with cold buffer (PBS-5% BSA) by centrifugation at 400×*g* for 10 min at 20°C. Cell pellets were resuspended in 100 µL PBS-BSA buffer and reacted with FITC anti-CD8 plus PE-Cy5 anti-CD4 mAb or FITC anti-CD14 for surface labeling. After 30 min of incubation in the dark at 4°C, the samples were washed three times in buffer by centrifugation at 300×*g* for 5 min at 20°C and fixed and permeabilized with freshly prepared Fixation/Permeabilization Working Solution (Pharmingen) for 30 min at 4°C, followed by two washed in 2 mL buffer. After the last wash, the cell pellets were resuspended in 100 µL 1X Permeabilization Buffer and reacted with PE anti-TNF-alpha at 4°C in the dark. After 30 min of incubation, the cells were washed twice with 2 mL 1X Permeabilization Buffer and then resuspended in 1% paraformaldehyde in Dulbecco's PBS (Sigma) for analysis. A total of 20,000 events/tube were acquired using a FACScalibur® flow cytometer (Becton Dickson). The Cell Quest™ software provided by the manufacturer was used for data acquisition and analysis.

### Statistical analysis

Genotype and allele frequencies were analyzed statistically by the chi-square test. The strength of association was estimated using odds ratios and 95% confidence intervals (CI). The Mann-Whitney or Kruskal-Wallis test was used to analyze the association of TNF-alpha levels with genotypes, allele frequency, serology, and clinical forms. The unpaired t-test was used to analyze the association of intracellular TNF-alpha expression with the cardiac or indeterminate form. Analysis was performed using the StatView software, version 4.57 (Abacus Concepts, USA). The level of significance was set at 5% (p<0.05).

## Results

### Characteristics of the sample

A total of 300 individuals were included in the study, 168 (56%) with positive serology for *T. cruzi* and 132 (44%) with negative serology. All subjects were from the same endemic area. A total of 214 subjects were genotyped to position -238. Of these, 100 individuals were seropositive for *T. cruzi* and 114 were seronegative. All 300 individuals were genotyped to position -308. Of these, 168 individuals were seropositive for *T. cruzi* and 132 were seronegative.

Clinical classification was possible in 119 of the 168 subjects infected with *T. cruzi*. Sixty-six (55.46%) had the cardiac form and 53 (44.54%) had the indeterminate form. Only 39 and 66 patients with the cardiac form were genotyped to positions -238 and -308, respectively. Among the indeterminate patients, 36 were genotyped to position -238 and 53 to position -308. Clinical manifestations were only compared between patients with the cardiac and indeterminate forms.

### TNF-alpha production

**Figure 1:**
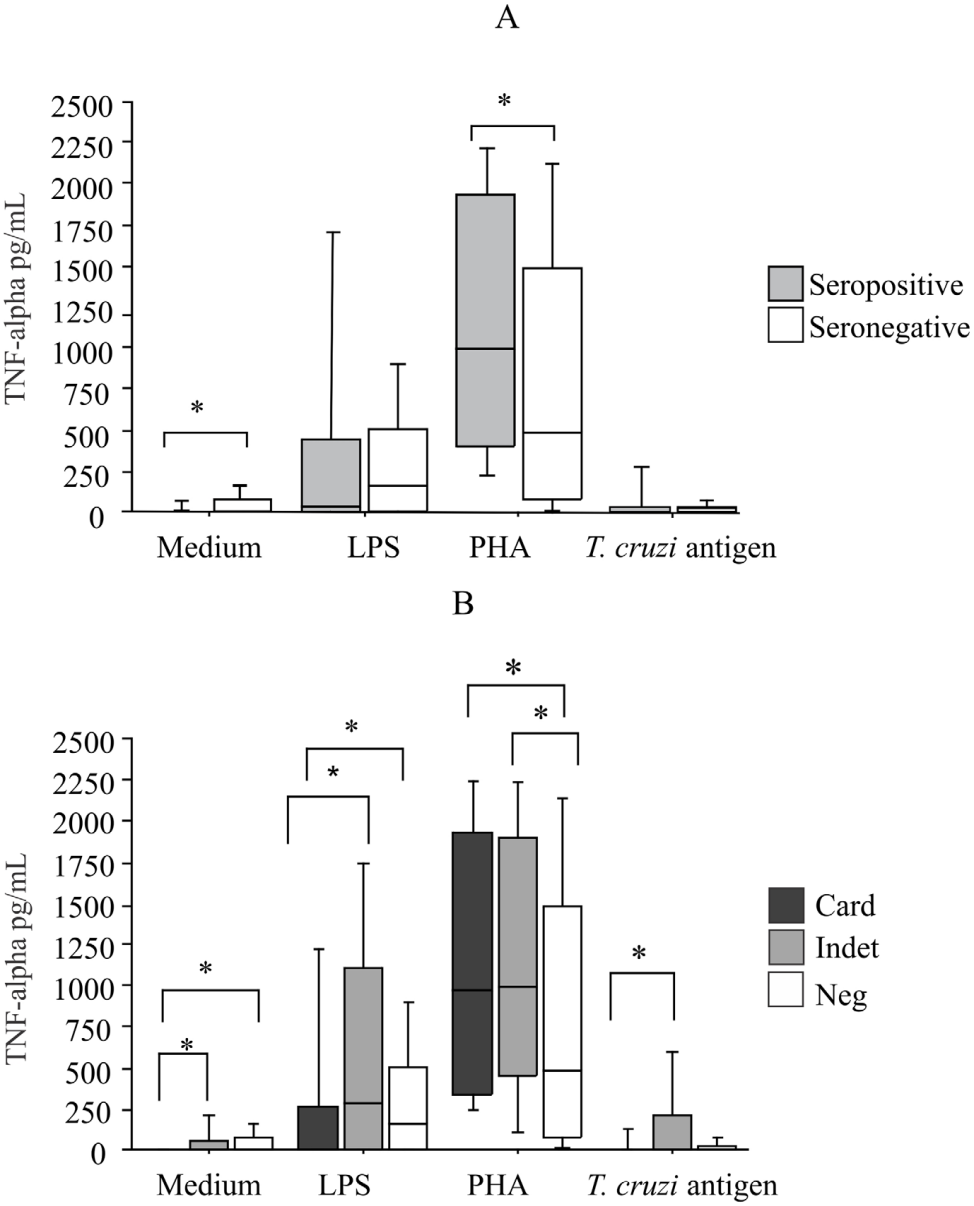
Levels of TNF-alpha in culture supernatants. TNF-alpha was assayed by ELISA in supernatants of PBMC cultured for 48 h in the absence of any stimulus and in the presence of *T. cruzi* antigen, LPS and PHA. **A.** Subjects were grouped according to positive or negative serology; *p = 0.0003 (Mann-Whitney test). **B.** Subjects were grouped according to the cardiac or indeterminate form and negative serology: Cardiac x negative to medium: p = 0.0002; cardiac x negative to LPS stimulation: p = 0.005; indeterminate x negative to PHA: p = 0.03; cardiac x indeterminate to medium: p = 0.01; to LPS: p = 0.005; to *T. cruzi* antigens: p = 0.01 (Mann-Whitney test). Results are expressed as pg/mL. The horizontal line indicates the median, bars the 25% and 75% percentiles, and vertical lines the 10% and 90% percentiles.

Seronegative individuals produced higher levels of TNF-alpha than seropositive subjects without stimulation (Mann-Whitney, p = 0.0003) and seropositive individuals produced more TNF-alpha than seronegative individuals after PHA stimulation (Mann-Whitney, p = 0.01) (**Figure 1A**). TNF-alpha levels were significantly higher in patients with the indeterminate form than in cardiac patients without stimulation (Mann-Whitney, p = 0.01), after LPS stimulation (Mann-Whitney, p = 0.005), and after stimulation with *T. cruzi* antigen (Mann-Whitney, p = 0.01) (**Figure 1B**). Comparison of seronegative individuals with cardiac and indeterminate patients showed that cardiac patients produced higher levels of TNF-alpha than seronegative individuals without stimulation (Mann-Whitney, p = 0.0002) and after LPS stimulation (Mann-Whitney, p = 0.005) (**Figure 1B**). Moreover, indeterminate patients produced higher levels of TNF-alpha than seronegative individuals after PHA stimulation (Mann-Whitney, p = 0.03) (**Figure 1B**).

### Association of TNF-alpha polymorphisms with disease

Analysis was performed between genotypes and infection determined by serology. **Table 1** shows the distribution of the TNF-238G/A and TNF-308G/A genotypes. No association was observed between genotypes and serology (*X^2^*, TNF-238G/A p = 0.08; TNF-308G/A p = 0.41). When the TNF-238AA and TNF-238GA genotypes were grouped as allele A presence and the genotype TNF-238GG was considered to be allele A absence, the data verified that allele A absence was more frequent among seronegative individuals (*X^2^*, p = 0.03) (**Table 2**). An odds ratio of 1.846 was observed (CI = 1.057 to 3.223).

**Table 1:** TNF-alpha genotypes found in subjects from an endemic area according to the presence or absence of infection.

	Seropositive individuals		Seronegative individuals		p value (X^2^)
Genotype	N	%	N	%	
TNF-238AA	26	26	22	19.30	
TNF-238GA	20	20	14	12.3	p = 0.08
TNF-238GG	54	54	78	68.4	
Total	100	100	114	100.0	
TNF-308AA	06	3.6	08	6.1	
TNF-308GA	52	31.0	34	25.8	p = 0.41
TNF-308GG	110	65.4	90	68.1	
Total	168	100.0	132	100.0	

**Table 2:** Presence and absence of the TNF-238A allele in seropositive and seronegative individuals.

	Seropositive individuals		Seronegative individuals		p value (X^2^)
	N	%	N	%	
Absence of TNF-238A allele	54	54.0	78	68.4	p = 0.03
Presence of TNF-238A allele	46	46.0	36	31.6	
Total	100	100.0	114	100.0	

**Table 3** shows the distribution of the TNF-238G/A and TNF-308G/A genotypes only among individuals with positive serology divided into the cardiac and indeterminate clinical forms. No significant associations were observed (*X^2^*, TNF-238G/A p = 0.28; TNF-308G/A p = 0.64).

**Table 3:** TNF-alpha genotypes found in subjects from an endemic area according to clinical form.

	Cardiac form		Indeterminate form		p value (X^2^)
Genotype	N	%	N	%	
TNF-238AA	10	25.6	8	22.2	
TNF-238GA	6	15.4	11	30.5	p = 0.28
TNF-238GG	23	59.0	17	47.3	
TOTAL	39	100.0	36	100.0	
TNF-308AA	4	6.1	2	3.8	
TNF-308GA	21	31.8	14	26.4	p = 0.64
TNF-308GG	41	62.1	37	69.8	
TOTAL	66	100.0	53	100.0	

### TNF-alpha production and genotypes

**Figure 2:**
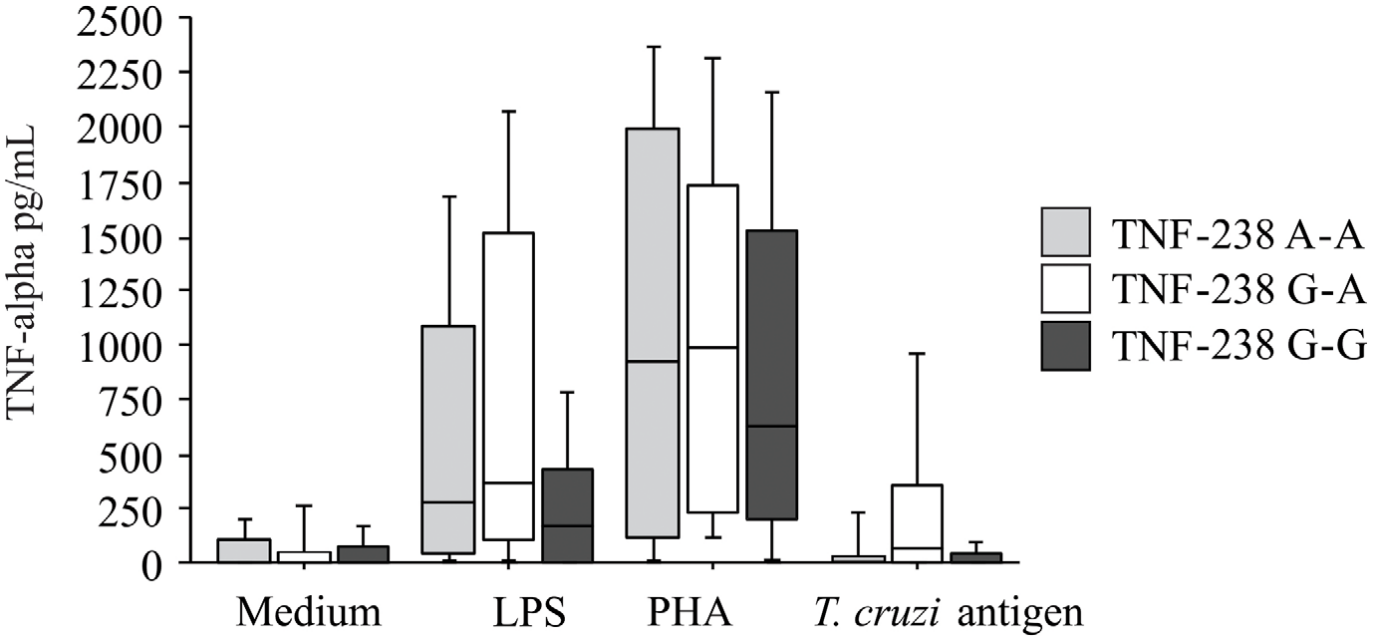
Levels of TNF-alpha in culture supernatants. TNF-alpha was assayed by ELISA in supernatants of PBMC cultured for 48 h in the absence of any stimulus and in the presence of *T. cruzi* antigen, LPS and PHA. Subjects were grouped according to TNF-238A genotypes (TNF-238AA, TNF-238GA and TNF-238GG). Individuals with positive and negative serology were analyzed. Results are expressed as pg/mL. The horizontal line indicates the median, bars the 25% and 75% percentiles, and vertical lines the 10% and 90% percentiles.

**Figure 3:**
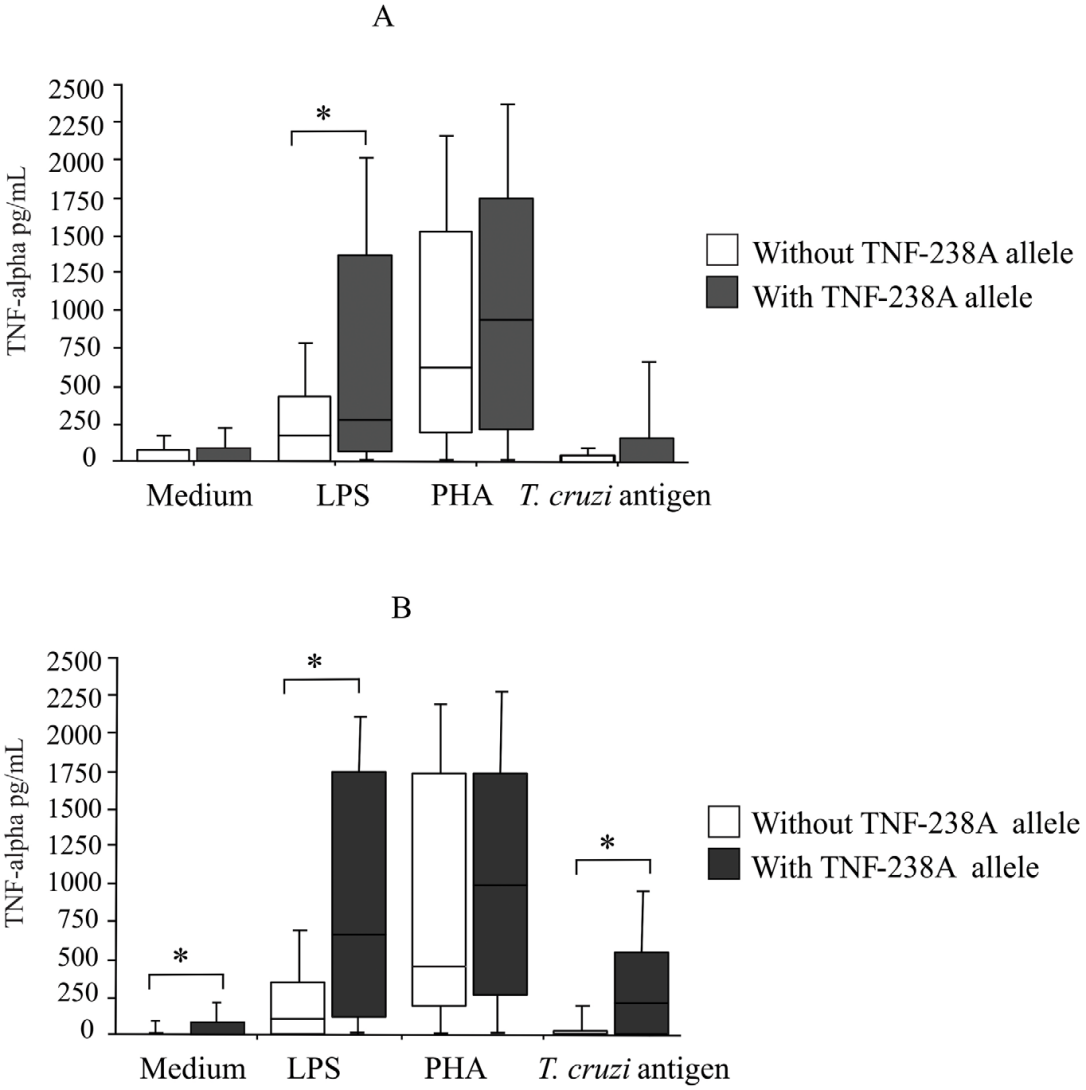
Levels of TNF-alpha in culture supernatants. TNF-alpha was assayed by ELISA in supernatants of PBMC cultured for 48 h in the absence of any stimulus and in the presence of *T. cruzi* antigen, LPS and PHA. **A.** Subjects with positive serology and negative serology were grouped according to the presence or absence of the TNF-238A allele; *p = 0.045 (Mann-Whitney test). **B.** Only subjects with positive serology were grouped according to the presence or absence of the TNF-238A allele; * p = 0.04 compared to medium, p = 0.004 compared to *T. cruzi* antigen, and p = 0.007 compared to LPS (Mann-Whitney test). Results are expressed as pg/mL. The horizontal line indicates the median, bars the 25% and 75% percentiles, and vertical lines the 10% and 90% percentiles.

There were no differences in TNF-alpha levels produced by seropositive and seronegative individuals carrying different genotypes of the TNF-alpha polymorphisms position -238 (**Figure 2**) and position -308 (data not shown). On the other hand, higher levels of TNF-alpha were observed in individuals carrying the TNF-238A allele after LPS stimulation (Mann-Whitney, p = 0.045) when all subjects were analyzed together (**Figure 3A**). No differences were observed when the individuals were grouped according to negative serology (data not shown). Interestingly, when seropositive individuals were analyzed alone, higher TNF-alpha levels were observed in those carrying the TNF-238A allele without stimulation (Mann-Whitney, p = 0.04), after stimulation with *T. cruzi* antigen (Mann-Whitney, p = 0.004), and LPS stimulation (Mann-Whitney, p = 0.007) (**Figure 3B**).

### Characterization of TNF-alpha-producing cells

**Figure 4:**
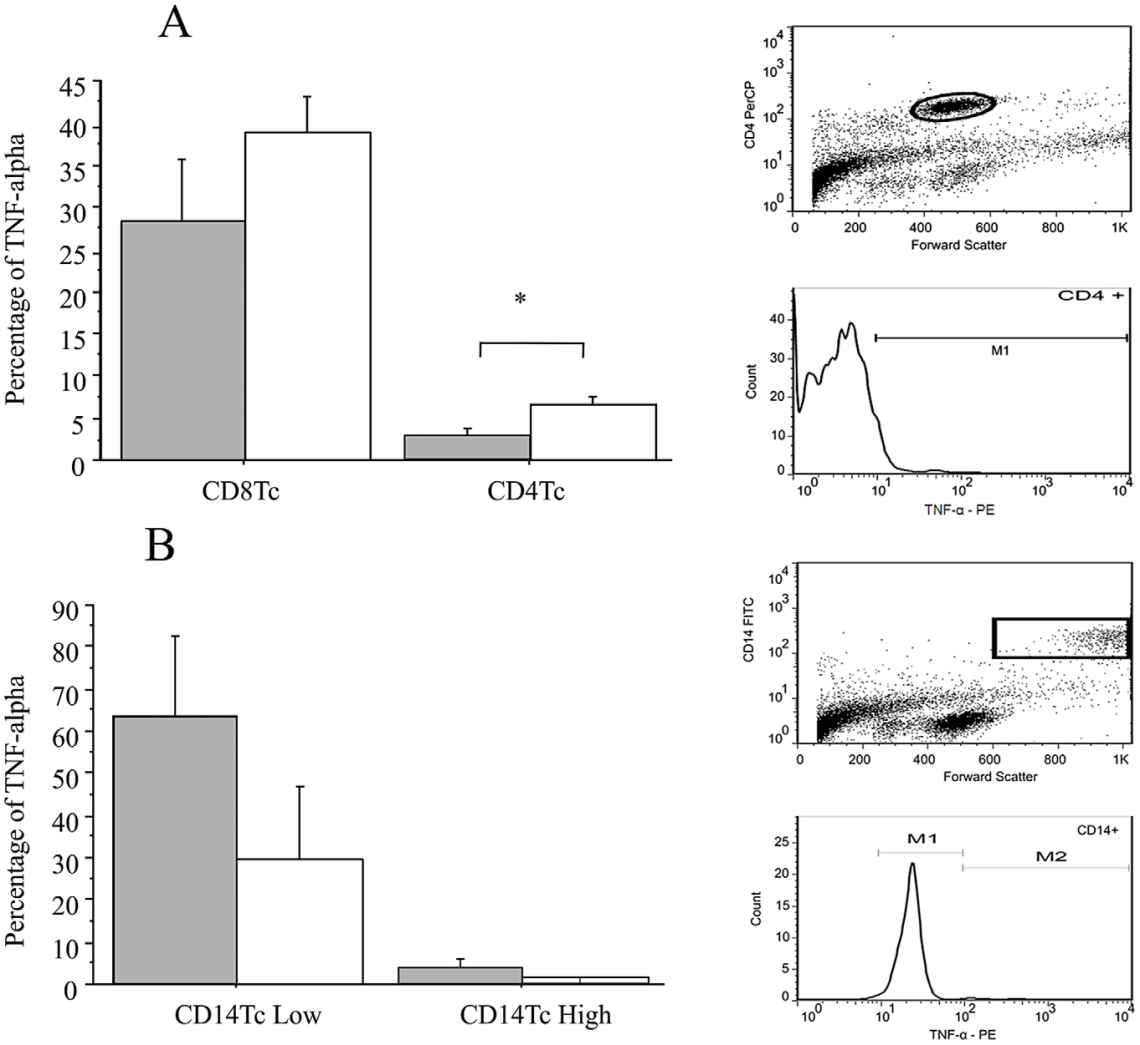
TNF-alpha expression on CD8^+^ and CD4^+^ lymphocytes. **A.** Percentage of TNF-alpha-producing cells among the CD8^+^ and CD4^+^ population of patients with the cardiac (gray bars) and indeterminate form (white bars) after 48 h of culture in the presence of *T. cruzi* antigens. **B.** Percentage of TNF-alpha-producing cells among the CD14^+^ population of patients with the cardiac (gray bars) and indeterminate form (white bars) after 48 h of culture in the presence of *T. cruzi* antigens. For analysis, a gate was established using FSC and surface marker flowed by analysis of intracellular TNF-alpha expression.

The presence of intracellular TNF-alpha was analyzed by flow cytometry in 48-h cultured cells. In the absence of stimulation, 1.18% and 1.83% of CD8^+^ cells were positive for TNF-alpha in patients with the cardiac and indeterminate forms, respectively. In CD4^+^ cells, the rate of TNF-alpha production was 2.60% in cardiac patients and 1.713% in indeterminate patients. No association was observed between the cell type and clinical form presented by the patient (data not shown). After stimulation with *T. cruzi* antigens, 28.47% and 38.80% of CD8^+^ cells were positive for TNF-alpha in patients with the cardiac and indeterminate forms, respectively. In CD4^+^ cells, positivity for TNF-alpha was 2.78% for cardiac patients and 6.54% for indeterminate patients (**Figure 4**). This difference was statistically significant (unpaired t-test; p = 0.044). Two levels of TNF-alpha production were observed in CD14^+^ cells, low and high levels. In cardiac patients, 63.36% and 3.76% of CD14^+^ cells were classified as low and high producers, respectively. These percentages were 29.96% and 0.70% in patients with the indeterminate form (**Figure 4**).

## Discussion

The immune response plays an important role in the control of *T. cruzi* infection. TNF-alpha is an important cytokine involved in parasite control during the acute phase [9]–[11]. However, there are few studies in the literature discussing its role during the chronic phase. Some studies have suggested the involvement of TNF-alpha in the development of the cardiac form [13], [29], [30], but its role in the control of parasite growth in humans and the consequent development of specific antibodies has been little studied. The present study conducted on individuals (control group and infected individuals) from an endemic area in the central region of Brazil (Água Comprida) provided important results. This area was included in the first national campaign against Chagas disease that started in 1950 [31]. These characteristics result in homogeneous population exposition and similar environmental and social conditions, thus reducing possible confounding factors.

Higher TNF-alpha levels were produced by seronegative individuals without stimulation, indicating that individuals who did not acquire the infection are able to produce TNF-alpha spontaneously. High basal levels of TNF-alpha may improve the host defense against *T. cruzi*, possibly by modulating the expression of iNOS and adhesion molecules involved in rolling, adhesion and extravasation during inflammatory events in response to *T. cruzi* invasion [10]–[12]. Higher levels of TNF-alpha were observed in seropositive individuals compared to seronegative subjects. This finding might be due to polyclonal expansion in response to nonspecific stimulation.

Analyzing the development of the clinical form, TNF-alpha levels were significantly higher in patients with the indeterminate form than in those with the cardiac form. Comparison of cardiac patients with the control group showed higher levels of TNF-α in the cardiac group without stimulation and after LPS stimulation. Furthermore, indeterminate patients produced higher level of the cytokine after PHA stimulation. This fact suggests that TNF-alpha production by PBMC might be more important for the ability of the cytokine to control parasite growth than for promoting tissue damage or the development of heart lesions. It is important to point out that in the present study most patients classified as having the cardiac form did not exhibit severe heart involvement such as heart failure. Severe and end-stage Chagas disease has been associated with high levels of TNF-alpha [29], [30], [32]. Our results differ from those reported by Ferreira et al. [29] and Talvani et al. [30]. However, a previous study from our group analyzing plasma TNF-alpha levels was unable to show differences between the clinical forms of chronic Chagas disease [33]. TNF-alpha plays a potential dual role, controlling parasite growth or promoting tissue damage. Moreover, TNF-alpha is able to stimulate IL-10 synthesis [34], a regulatory cytokine that contributes to the control of inflammation. A recent experimental study demonstrated that the blockage of TNF-alpha with Etanercept enhances left ventricular dysfunction in *T. cruzi*-induced chronic cardiomyopathy [35]. In this respect, the delicate balance between the ability of TNF-alpha to control parasite growth and to promote tissue damage may be responsible for human resistance/susceptibility to Chagas disease. Not only the level of this cytokine, but the cells involved in its production and the elapsed time after interaction with the parasite should be investigated to improve the current understanding of the role of TNF-alpha in Chagas disease.

Regarding TNF-alpha gene polymorphisms, this study provided evidence of an association between the absence of allele A at position -238 and seronegativity. We observed that individuals carrying the TNF-238A allele produce higher levels of TNF-α than those without the allele. This result suggests that the TNF-alpha polymorphism affects gene expression and that this effect depends on the cell population and on the strength of the stimulus since no differences were observed after PHA stimulation. Moreover, infected individuals carrying the TNF-238A allele produced higher levels of TNF-alpha than those without allele A. This result suggests that the -238 polymorphism exerts its potential function in infected subjects, probably as a result of clonal expansion and immunoregulatory mechanisms established during infection. The effect of the polymorphism might be more pronounced under these conditions. No significant association was established for the -308 polymorphism.

A higher proportion of the TNF-238A allele among patients when compared to controls was demonstrated for various other infectious diseases such as infection with *Chlamydia trachomatis*, although these findings were not statistically significant [36]. Another study reported a higher bacteriological index in patients with leprosy carrying the TNF-238A allele [37]. Homozygous carriers of the TNF-238A allele were more frequent among patients with psoriasis compared to controls [17]. In chronic and active hepatitis C, the TNF-238A allele was more frequent in the group of patients than in controls [18].

Other studies on Chagas disease were conducted in Peru, Mexico and Brazil. The first study analyzed 87 healthy controls and 85 individuals seropositive for anti-*T. cruzi* antibodies from an endemic area in Peru. No significant differences were observed between patients and controls or between asymptomatic individuals and patients with cardiomyopathy for either polymorphic region [19]. The second study compared 54 individuals seropositive for Chagas disease and 169 controls from the Mexican population. A higher frequency of the TNF-308A allele was observed in chagasic patients compared to the control group. Furthermore, the TNF-308A allele was more frequent among patients with cardiopathy compared to asymptomatic individuals [20]. However, these differences in the results might be attributed to ethnic variations among different populations and to differences in the study design and patient selection criteria. The third study analyzed 42 patients with severe ventricular dysfunction. Patients carrying the TNF-308A allele presented a significantly shorter survival time than those carrying other alleles [21]. The same group compared 166 chronic Chagas disease patients with cardiomyopathy to 80 asymptomatic patients, but observed no significant association with TNF polymorphisms [22]. However, none of the studies conducted in Brazil investigated the -238 gene polymorphism or included subjects from endemic areas.

The present study differs from previous investigations because all individuals were from a region of high endemicity, with a greater probability of a more homogenous condition of exposure and accurate clinical analysis after some years of infection, permitting a sufficient time interval for the development of the clinical forms of the disease. This is advantageous in relation to the study's characteristics, though it represents a disadvantage due to the possible death of patients with more severe forms of the disease. Furthermore, in the present study, functional analysis showed that PBMC from seropositive individuals carrying the TNF-238A allele produced significantly higher levels of TNF-alpha when stimulated with LPS and *T. cruzi* antigens and in the absence of any stimulus.

Other investigators observed higher TNF-alpha production by LPS-stimulated PBMC in individuals homozygous for the TNF-308A allele [38]. Studies using gene construction strategies in which the promoter region of TNF was ligated to the luciferase gene have also shown an increased production of TNF-alpha levels among individuals with the TNF-308A allele [39], [40]. A repressor-binding site was identified at position -238 [41]. Thus, it is possible that in certain cell types, the presence of the polymorphism reduces affinity for the repressor, with a consequent increase in transcription [42]. These data indicate that the polymorphism at this position may influence gene expression and may help explain the association between the presence of allele TNF-238A and higher TNF-alpha production observed in this study.

Flow cytometry analysis indicated that CD14^+^ and CD8^+^ cells are the major source of TNF-alpha after antigen stimulation. The number of CD14^+^ T lymphocytes expressing TNF-alpha was significantly higher in cardiac patients, whereas the number of CD4^+^ lymphocytes producing this cytokine was high in patients with the indeterminate form. The stimulation of CD8^+^ T lymphocytes with exogenous antigens, including *T cruzi* lysate antigens, under similar culture conditions has been demonstrated previously [43], [44].

The importance of TNF-alpha as an activator of the mechanisms involved in parasite elimination [12] and in tissue injury [10], [11] has been clearly demonstrated in experimental models by biological blockage of the cytokine or in genetically modified animals. Studies on humans have associated TNF-alpha with phenomena of tissue injury during the chronic phase of infection [13]. The present results demonstrate an association between gene polymorphisms and infection.

In conclusion, the present results suggest that the TNF-238A allele exerts a significant effect on human susceptibility to infection. Furthermore, seropositive individuals carrying the TNF-238A allele produce higher levels of TNF-alpha after antigen and polyclonal stimulation, suggesting that the presence of the allele is associated with higher TNF-alphaproductionIn addition to its trypanocidal activity, TNF-alpha seems to trigger regulatory networks, such as the induction of IL-10 [34], that permit parasite escape. The development of clinical forms of Chagas disease may implicate other genes involved in the delicate balance of immune response.
